# Supplementary material for: Worker Protection Scenarios for General Analytical Testing Facility under Several Infection Propagation Risks: Scoping Review, Epidemiological Model and ISO 31000
Source: Int J Environ Res Public Health. 2022 Sep 22;19(19):12001. doi: 10.3390/ijerph191912001 (PMC9565149; doi:10.3390/ijerph191912001)
Supplement: Supplementary file 1 [file ijerph-19-12001-s001.zip › ijerph-1915421-supplementary.pdf]

International Journal of
Environmental Research and Public Health
MDPI

Supplementary Materials S1. Review of the transmission modes of causative agents of gastrointestinal diseases to identify possible direct, fomite-mediated, or surface-mediated transmission

○ **Enterohemorrhagic *Escherichia coli* (EHEC)**

The possibility of indirect transmission through fomites (toys) at home environment and children's daycare centers has been reported [29]. Human-to-human transmission was also reported at day care facilities for intellectual disabilities via fomites in places such as restrooms and on inorganic surfaces [30].

○ **Invasive non-typhoidal Salmonellosis (iNTS) species**

Salmonella typhimurium has been reported to be transmitted directly from person to person in an ecologically segregated healthy population (within an Indian reserve area) [44]. In this case, there was also no common exposure such as water and food, and the case of hospitalization due to the primary case of digestive infection was excluded.

○ ***S. typhimurium***

It is a model microorganism for studying surface or fomite mediated transmission under mechanical stress or low water activity condition, It is reported that the virulence factor is rather increased by viability in fomites [60]. Palmer et al. reported cases of indirect propagation of *Salmonella typhimurium through* fomites at a university facility [45]. Meanwhile, in the case of the invasive typhoidal *Salmonella*, the major transmission route of *S. typhi* was found to be the "four Fs" (flies, fingers, feces, and fomites) [53]. Most of the transmission cases are through direct fecal-oral routes through contaminated common water or fomites contaminated by infected cases. The possibility of propagation via fomites increases as the density of the population increases.

○ ***Shigella dysenteriae***

There are no reports of spread within the health group according to result of the scoping review. However, the possibility of maintaining virulence and their survival in fomites or inorganic/organic surfaces has been reported [55]. Since this strain is well known for person-to-person contact transmission and indirect transmission [66], it is believed that there is a possibility of indirect transmission through the sharing of objects and environments within the health group.

○ ***Vibrio* species**

This species has been reported not only in cases of infection through food and water but also in the possibility of direct transmission through the direct fecal-oral route [64]. In addition, the direct and indirect transmission by the bacillary shedding of infected cases was confirmed in a domestic environment [47]. Furthermore, it has been reported that a variable cell state can be maintained for 1–2 days on dry surfaces such as aluminum, metal, coins, paper, plastic, and silk [59]. Even if it belongs to the same genera, *V. parahemolyticus*, there are no studies that provide evidence for the possibility of person-to-person transmission. No literature related to indirect transmission via fomites or surface has also been reported.

○ **Enteric viruses**

Transmission within the healthy population in the social group (e.g. school, financial institutions, residential, vehicle, olympic games etc.) has been problematic periodically in the Korean society, and direct and indirect transmission between non-co-exposure sources is reported at a high frequency. Direct or fomite-mediated transmission cases of enterovirus and norovirus in the indoor environment (homes, offices, schools, workplaces, transport systems, airplane, ships) [38, 39, 42, 48] have been reported frequently and constantly. In particular, the spread within vehicles such as ships and airplanes showed that despite the short contact time, the transmission rate due to the indirect contact of norovirus was very fast [48]. In addition, the direct or indirect transmission of norovirus was reported through the use of aerosols [40], and indirect transmission occurred between healthy soldiers through fomites in the military base [41, 50]. In such

cases of indirect transmission within such isolated social groups, it was found not to be food or waterborne [35]. Person-to-person transmission of hepatitis A was confirmed in a geographically isolated population without co-exposure to water or food [51]. The primary case that was infected with hepatitis A virus through food consumption who attended school and spread the infection to all students was reported [43]. Sattar reported fomite-mediated transmission of rotavirus in the Indian language society, whereas Butz reported this occurrence at a daycare center [34, 46]. The possibility of spreading various organic and inorganic fomites of enteric viruses has also been reported in diverse studies [56, 58, 61].

○ **Gram-positive or spore-forming bacterial species**

Person-to-person transmission has not been highlighted historically as a major issue because the genus *Staphylococcus* sp. causes a mass poisoning outbreak by toxins that proliferate in food. As is commonly known, the long-term survival in various agents through environmental resistance was investigated [36, 55], and given its significant distribution in health groups [32], the propagation potential was confirmed in general office or laboratory environments. However, this view was related to the spread of a multi-drug resistant *Staphylococcus aureus* that is problematic socially. Moreover, *Clostridium perfringens* causes food poisoning and is transmissible via the direct fecal-oral route [37]. Since *S. aureus* and *C. perfringens* are part of the human normal flora in a wide population [18, 82] and the management of toxin production is important, it is not meaningful to develop a management plan according to the propagation route. Moreover, most people have antibodies against *C. perfringens* enterotoxin (CPE), i.e., most populations have been exposed to this pathogen at least once. The excretion of *C. perfringens* in stool samples in healthy people is not uncommon [37, 62]. Although the transmission of vegetative cells does not occur due to their anaerobic properties, it may cause environmental pollution of endospores in large quantities from carrier or apparent infection patients. However, environmental hygiene management in the workplace is more important. Therefore, considering the route of transmission, only the quarantine of active patients is necessary, and no other measures are needed.

○ ***Giardia intestinalis***

It is water or foodborne, and compared with enteric viruses and gastrointestinal bacteria, its ability to withstand on inorganic/organic fomites has been reported due to its strong environmental and disinfectant resistance, with the fomites being a major route of propagation. The path of direct transmission is limited, and it is a case of transmission due to contact with the patient's fecal contaminated hand [68, 70].

○ ***Entamoeba* species**

The direct person-to-person transmission of protozoan intestinal infectious diseases by *Entamoeba histolytica, coli* is not common except in areas with extreme poor hygiene conditions. Rather, transmission occurs while secreted by patients and it remains in the environment. Patients are infected with the dysentery amoeba send non-infectious trophozoite and infectious cyst through their feces. The trophozoite finds it difficult to withstand in the external environment and stomach acid, and direct transmission is only possible through sexual contact [71]. Therefore, the possibility of spreading protozoa through inorganic fomites is limited, as reported in experimental studies [69].
